# Unique expression signatures of circular RNAs in response to DNA tumor virus SV40 infection

**DOI:** 10.18632/oncotarget.21694

**Published:** 2017-10-09

**Authors:** Jiandong Shi, Ningzhu Hu, Jianfang Li, Zhaoping Zeng, Ling Mo, Jing Sun, Meini Wu, Yunzhang Hu

**Affiliations:** ^1^ Institute of Medical Biology, Chinese Academy of Medical Sciences, Peking Union Medical College, Kunming 650118, China; ^2^ Yunnan Key Laboratory of Vaccine Research and Development of Severe Infectious Disease, Kunming 650118, China; ^3^ Yunnan Provincial Key Laboratory of Vector-Borne Diseases Control and Research, Pu’er, Yunnan 665000, China

**Keywords:** circular RNA, SV40, carcinogenesis, transcriptome, Vero cells

## Abstract

Circular RNAs (circRNAs), identified as a class of widely expressed endogenous regulatory RNAs, are involved in diverse physiological and pathological processes. However, their role in viral pathogenesis and cellular antiviral response remains unexplored. In this study, a potent DNA tumor virus, simian virus 40 (SV40), was used as a model to investigate the viral influences on cellular circRNA transcriptome.

Using RNA-seq, 15,241 putative circRNAs were identified *de novo* from 5,057 parental genes in monkey kidney–derived Vero cells. The expression of selected circRNAs was confirmed by reverse transcription-polymerase chain reaction and Sanger sequencing. Further analysis showed that most circRNAs comprised multiple exons, and most parental genes produced multiple circRNA isoforms. A total of 134 significantly dysregulated circRNAs, including 103 upregulated and 31 downregulated circRNAs, were found after SV40 infection. Gene ontology and Kyoto Encyclopedia of Genes and Genomes pathway analysis revealed that various cancer-related pathways, including p53 and Wnt pathway, could be affected by SV40 infection via the alteration of the circRNA hosting genes. Moreover, diverse cellular immune pathways, including Toll-like receptor pathway and Janus kinase–signal transducer and activator of transcription pathway, could also be affected by SV40 infection. An integrated circRNA-miRNA-gene analysis suggested the putative function of circRNAs as cellular and viral miRNA decoys to indirectly regulate the gene expression during SV40 infection. This study presented the first comprehensive expression and functional profile of circRNAs in response to SV40 infection, thus providing new insights into the mechanisms of viral pathogenesis and cellular immune response.

## INTRODUCTION

Simian virus 40 (SV40), a polyomavirus of rhesus macaques was identified in 1960 [1]. After its discovery, SV40 was found to induce tumors in animals and transform a variety of cell types from different species, and was also implicated in the development of several human cancers [2, 3, 4]. Circular RNAs (circRNAs), a new type of endogenous regulatory RNAs, have been identified in a variety of tumors [5, 6, 7]. However, whether circRNAs are involved in viral pathogenicity and host–virus interactions remains unclear.

CircRNAs, produced from the backsplicing of pre-mRNAs, do not possess a covalently closed circular structure with neither 5′–3′ polarities nor polyadenylated tails, increasing their resistance to exonuclease or RNase R compared with their linear counterparts [8, 9]. An enormous variety of endogenous circRNAs, such as exonic circRNA (ecircRNA) [10, 11], intronic circRNA (ciRNA) [12, 13], and exon–intron circRNA (EIciRNA) [14], arises from different genomic regions of eukaryotic cells. However, a vast number of circRNAs are derived from the eukaryotic exonic regions of pre-mRNAs via the backsplicing process, which joins the downstream splice donor site (5′ splice site) to an upstream acceptor splice site (3′ splice site) in a “head-to-tail” splicing manner [15]. Although circRNAs were first discovered in the early 1990s, they were considered the products of aberrant RNA splicing because they could not be sequenced [10]. The development of RNA-sequencing (RNA-seq) technology has allowed the rediscovery of circRNAs, enabling the identification and characterization of a large number of circRNAs in recent studies. These identified circRNAs are endogenous, abundant, evolutionarily conserved, and present a tissue/developmental-stage- or cell-type-specific expression pattern [11, 16, 17]. Emerging evidence indicates that circRNAs can function as microRNA (miRNA) sponges or potent competing endogenous RNA (ceRNA) molecules [16, 18, 19, 20], regulators of splicing and transcription [15, 18], modifiers of parental gene expression [12, 14, 19], and templates for translation [21, 22]. Other recent studies have suggested that circRNAs may be involved in the initiation and development of cancer and neurodegenerative diseases, and can potentially become new diagnostic or predictive biomarkers of both physiological and pathological processes [23, 24, 25]. Therefore, as an emerging key epigenetic regulator in transcription and posttranscription, circRNA provides novel insights into the underlying mechanism of tumorigenesis and host–virus interactions. However, a majority of studies have focused only on linear RNAs, such as long noncoding RNAs (lncRNAs) and miRNAs, for their roles, in viral pathogenicity and host antiviral response. Whether circRNAs are involved in viral pathogenesis and host immune response is still poorly understood.

The DNA tumor virus SV40 has served as a powerful model to study the mechanisms underlying carcinogenesis and viral influences on cellular processes. The present study systematically investigated the remodeling effect of DNA tumor virus SV40 on cellular circRNA repertoire and identified a large number of dysregulated circRNAs during SV40 infection. The potential biological functions of dysregulated circRNAs associated with viral pathogenicity and host immune responses were revealed. Based on these results, the present study presented the first transcriptome-wide circRNA landscape in SV40-infected Vero cells, thus providing novel insights into the understanding of the mechanisms of viral carcinogenesis and cellular immune response.

## RESULTS

### Identification of circRNAs in SV40-infected and uninfected Vero cells

**Figure 1:**
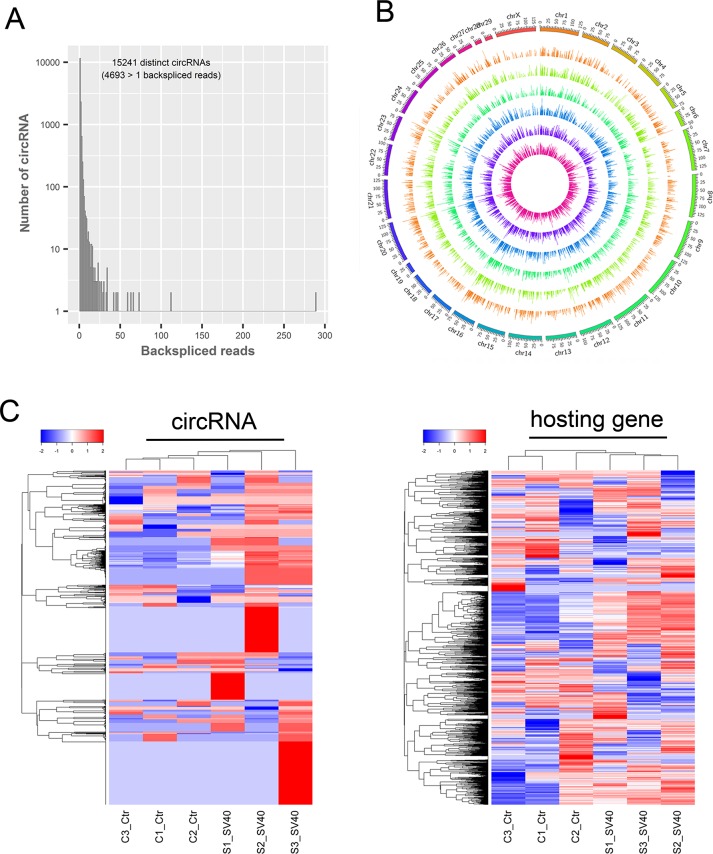
Transcriptome-wide identification of circRNAs in SV40-infected Vero cells **(A)** The number of circRNAs and back-spliced reads identified in six RNA libraries of Vero cells. **(B)** The overview of all the circRNA transcripts abundant on different chromosomes. All circRNA transcripts are presented based on their expression value (FPKM) by mapping them to the AGM reference genome. **(C)** A clustered heat map showing expression patterns of the most highly expressed circRNAs (left; FPKM >10) and their corresponding linear hosting mRNA transcripts (right). Color from blue to red; the deeper the color, the higher the expression.

In the RNA-seq-based identification, circRNAs were recognized based on backspliced reads. Backspliced reads refer to the RNA-seq reads that contain splice junction formed by joining a downstream splice donor to an upstream splice acceptor (head to tail). Thus, six rRNA-depleted RNA libraries were sequenced and computationally analyzed for screening backspliced reads to identify circRNAs in the African green monkey kidney (AGMK)-derived Vero cells. The reads were mapped to the AGM reference genome using TopHat-Fusion. The results showed that approximately 50% fusion reads were mapped to exons, approximately 42% to introns, and approximately 8% to intergenic regions. The genome-wide analysis of RNA-seq data revealed a large number of highly reliable backspliced reads that were recognized as circRNA candidates. These circRNA candidates, contained distinct types of circRNAs whose backsplice junctions were flanked by the GU/AG intron signal and were derived from exons (circRNA) and introns (ciRNA). Briefly, a total of 3822, 4099, and 3629 circRNAs were identified in three uninfected libraries. However, a total of 4171, 4334, and 4952 distinct circRNAs were identified in three SV40-infected libraries (Supplementary Table 1). The majority of circRNAs (15235/15241) possessed a small number of backspliced reads (<50 reads), thus suggesting their low abundance (Figure 1A). In total, using this *de novo* method, 15,241 unique circRNAs with a considerable number of isoforms were identified from 5,057 hosting genes. The total number of detected circRNAs in SV40-infected cells was slightly higher (10,096 vs 8,817) than that in the uninfected cells, which was perhaps due to the SV40 infection stress. Interestingly, most circRNAs were derived from exons of protein-coding genes, which comprised one or multiple exons and accounted for about 98% of total circRNAs. Intron-derived ciRNAs accounted only for about 2%. These characteristics were consistent with those of human circRNAs and implied their unique splicing patterns. Additionally, an overview of global circRNA abundances on different chromosomes was presented based on their expression value (fragments per kilobase of transcript per million or FPKM) by mapping all circRNA transcripts to AGM reference genome (Figure 1B). Clustering analysis of highly expressed circRNAs (FPKM > 10) and their parental genes suggested different expression patterns of circRNAs and their hosting genes (Figure 1C). Taken together, a large number of putative circRNAs from their hosting genes were *de novo* identified by RNA-seq, thus presenting a transcriptome-wide circRNA expression landscape in Vero cells.

### Validation of circRNAs by reverse transcription–polymerase chain reaction and Sanger sequencing

**Figure 2:**
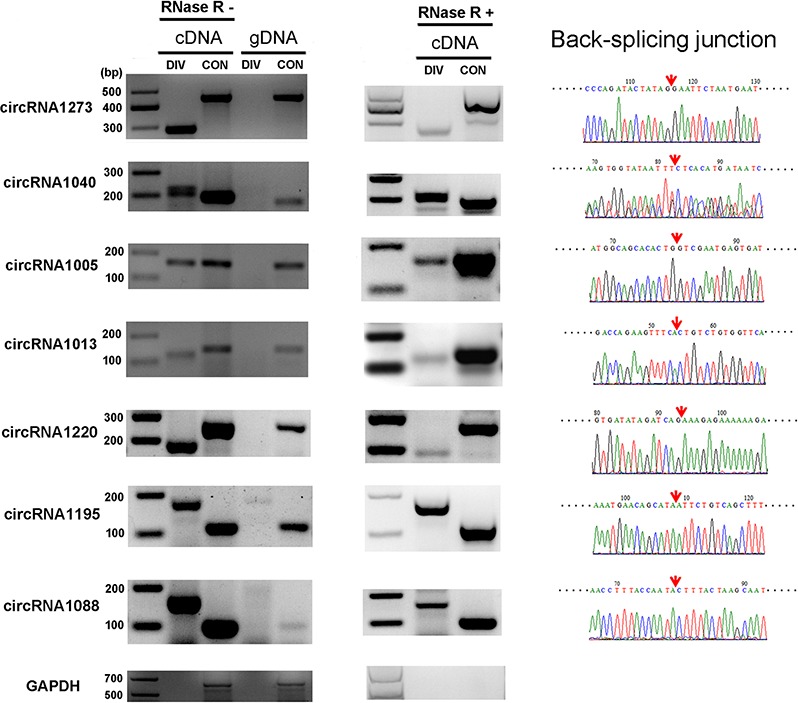
Verification of circRNAs using RT-PCR and Sanger sequencing Expression of seven selected circRNAs was validated by RT-PCR using divergent (DIV) primers covering the backsplice junction. The linear *GADPH* gene served as an internal control. The red arrow indicates the backsplicing site of circRNA.

Seven putative circRNA sequences were selected from the annotations to confirm the expression of the circRNA candidates. Moreover, a variety of experimental analyses including RNase R resistance analysis, reverse transcription–polymerase chain reaction (RT-PCR) and Sanger sequencing, were performed. The designed divergent and convergent PCR primers were used to amplify selected circRNA candidates in the total RNA, RNase R-treated RNA, and genomic DNA. As expected, the expression and backplicing junctions of the selected circRNAs, including circRNA1273, circRNA1040, circRNA1005, circRNA1013, circRNA1220, circRNA1195 and circRNA1088, were confirmed by RT-PCR in the total RNA and RNase R-treated RNA samples. Backsplicing junctions of circRNAs were amplified only in cDNA samples and further validated by Sanger sequencing (Figure 2). This result indicated that identification of circRNAs was reliable in the RNA-seq datasets based on the pipeline of CIRCexplorer [26].

### Relationship between circRNAs and their parental genes

**Figure 3:**
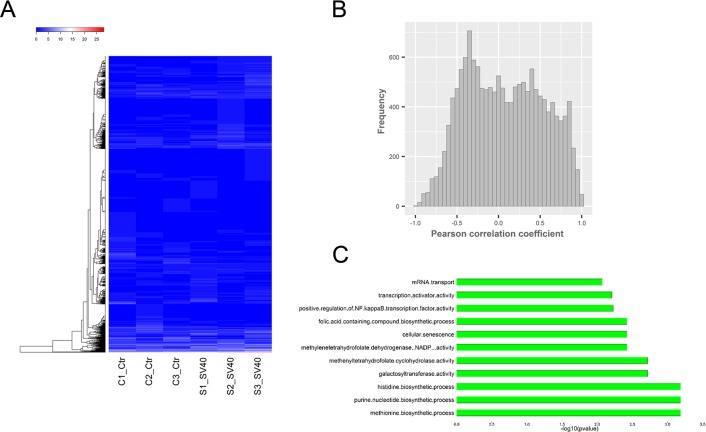
Relationship between circRNAs and their parental genes **(A)** Thecircularized levels of highly expressed circRNA hosting genes (FPKM >10) were similar in different samples. **(B)** The Pearson correlation analysis suggested a moderate co-expression pattern between circRNAs and their parental genes. **(C)** The most common circRNA hosting genes were revealed by the top 11 enriched GO terms.

Moreover, circularized levels of highly expressed circRNA hosting genes (FPKM >10) were similar in different samples (Figure 3A). A Pearson correlation analysis suggested a moderate correlation between the expression of circRNAs and their parental genes (Figure 3B). The results were consistent with the findings of previous studies [26, 27], thus suggesting the circularized patterns of parental genes. The annotation, expression, and isoforms of the circRNAs and the structural relationship with their parental genes and their flanking introns are shown in Supplementary Table 2. Additionally, approximately 2,286 and 2,559 hosting genes on an average in uninfected and SV40-infected libraries, were further enriched for their function by gene ontology (GO) and Kyoto Encyclopedia of Genes and Genomes (KEGG) analyses (Supplementary Table 2). The most common circRNA hosting genes, which were implicated in different molecular functions and biological processes, were revealed by top 11 enriched GO terms (Figure 3C). These results suggested a specific co-expression and circularized pattern between circRNAs and their parental genes.

### Genomic features of circRNAs in SV40-infected and uninfected Vero cells

**Figure 4:**
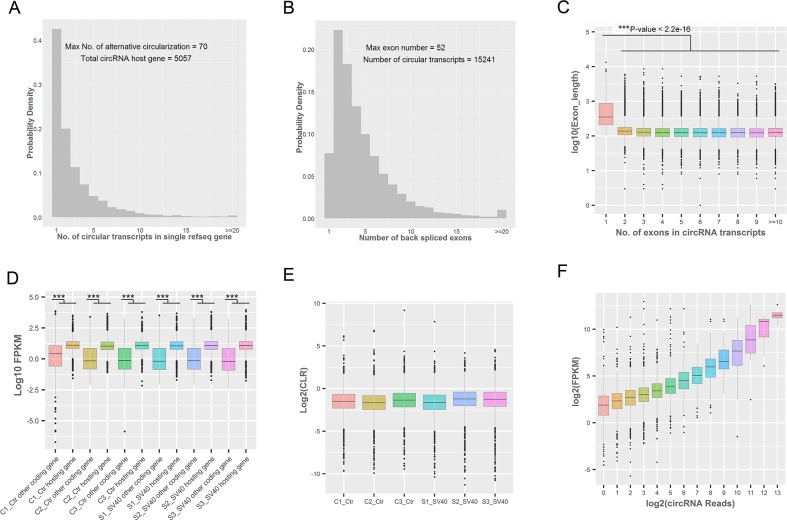
Genomic and expression characteristics of circRNAs **(A)** The distribution of the number of circRNA transcripts in a single circRNA hosting gene. **(B)** The distribution of the number of back-spliced exons in a single circRNA transcript. **(C)** The length distribution of back-spliced exons. The length of circRNAs with one exon was much longer than the length of circRNAs with multiple exons (^***^P value <0.001; *t* test). The box plots indicate the exon length distribution (*y*-axis) from circRNAs consisting of a different number of back-spliced exons (*x*-axis). **(D)** The comparison of the expression levels of circRNA hosting genes and other coding genes in each sample. The expression levels of circRNA hosting genes only containing linear transcripts; the expression levels were higher than those of other coding genes (^***^P value <0.001; *t* test). **(E)** The CLR values of circRNA hosting genes in each sample. The box plots indicate the CLR value in a log2 scale for circRNA hosting genes (*y*-axis) in different samples (*x*-axis). **(F)** The relationship between circRNA reads and FPKM of their hosting genes. The box plots indicate the FPKM value in log_2_ scale of circRNA hosting genes (*y*-axis) corresponding to different circRNA reads in the log2 scale (*x*-axis).

The distribution of the number of different types of circRNAs from each circRNA hosting gene showed that the vast majority of hosting genes produced multiple circRNA isoforms as “hot-spot” genes, and the maximum number of alternative circularization was 70 in a single RefSeq hosting gene (Figure 4A). Interestingly, the present study found that 15,227 (99.9 %) of 15,241 circRNAs possessed middle exons of their hosting genes, and were not associated with the first and last exons of their hosting genes (Supplementary Figure 1A), which was consistent with the findings of previous studies [26, 27]. A great majority of circRNAs (14063/15241; accounted for 92.27%) were composed of multiple exons, and the maximum number of exons in a circRNA was 52 (Figure 4B), which was consistent with the result of a previous study [27]. The length distribution of circRNAs showed that the length of the majority of ecircRNAs (11848/15241; accounted for 77.74%) was less than 1 KB (Supplementary Figure 1C). Additionally, most circRNAs (13026/15241; accounted for 85.47%) contained 2–10 exons, and about 7.73% of ecircRNAs contained only 1 exon (Supplementary Figure 1B). Notably, circRNAs with only one exon were much longer than circRNAs with multiple exons (*P* value < 2.2e-16; *t* test) (Figure 4C), consistent with previous reports [26, 28]. Yet, most identified circRNAs in the present study possessed more exons than circRNAs detected in previous studies [26, 29]. Further, the introns flanking the circRNAs were found to be slightly longer than the control introns (upstream flanking intron: median 5.646 KB; downstream flanking intron: median 5.344 KB; all introns: median 2.031 KB) (Supplementary Figure 1D), which was consistent with the findings of previous studies [26, 30, 31]. The distribution of flanking intron length of exon-derived circRNAs showed that the lower expression of circRNAs was also flanked by longer introns (Supplementary Figure 1E and 1F), suggesting that RNA circularization did not require long flanking introns. Moreover, the expression levels of circRNA hosting genes were compared with the expression levels of other coding genes that did not have detectable circular transcripts to further investigate the expression patterns of circRNAs. The results showed that the expression levels of circRNA hosting genes were higher than those of other coding genes in all samples (C1, *P* value = 0.009348; C2, *P* value < 2.2e-16; C3, *P* value = 8.757e-14; S1, *P* value = 6.127e-13; S2, *P* value = 6.417e-13; S3, *P* value = 1.056e-14) (Figure 4D), suggesting that these circRNA hosting genes were more resistant to the linear RNA decay machineries compared with the other coding linear transcripts. Further, the value of the circular to the linear ratio (CLR) [32] of the circRNA hosting genes in all samples suggested the relatively lower abundance (circRNA8070 vs its parental gene IGFBP3 in PFKM: 2674.58 vs 6133.93) of a given circRNA compared with its linear transcript (Figure 4E). In addition, the relationship between circRNA reads and their hosting gene expression was analyzed. The results indicated that genes with a higher number of circRNA reads also showed higher expression levels of their linear transcripts (Figure 4F). The detailed analysis might imply that the regulations and production mechanisms for alternative circularization in Vero cells are rather complex and require further systematic investigation.

### Differential expression analysis of circRNAs in response to SV40 infection

**Figure 5:**
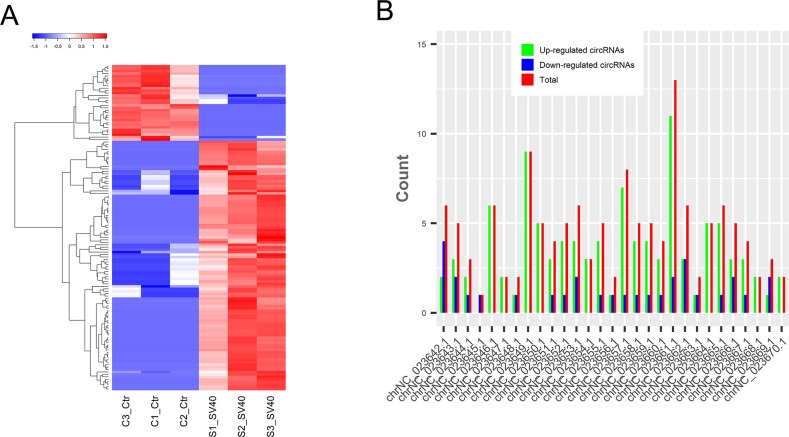
Dysregulated circRNAs in response to SV40 infection **(A)** The clustered heatmap of differentially expressed circRNAs based on their FPKM value. Red color indicates high expression level, and blue color indicates low expression level. **(B)** The distribution of differentially expressed circRNAs on different chromosomes of the AGM.

The global circRNA expression profiles were compared between SV40-infected and uninfected cells to gain insight into the expression pattern and function of the circRNAs. The results showed that 134 circRNAs were significantly dysregulated (log_2_ fold change ≥1 or ≤ -1 and *P* < 0.05); of these, 103 circRNAs were upregulated while 31 were downregulated (Supplementary Table 3). More upregulated circRNAs suggested their dominant role in host–virus interactions. The cluster analysis indicated that circRNAs were more highly expressed (10,096 vs 8,817) in SV40-infected cells than in uninfected cells (Figure 5A). Moreover, they were distributed on all chromosomes of Vero cells (Figure 5B). Notably, more than 100 exon-derived circRNAs were significantly upregulated and 31 were downregulated. In contrast, the intron-derived circRNAs contained only upregulated circRNAs (Supplementary Table 3). These results suggested that ecircRNAs might play a dominating role in regulating cellular response to virus infection.

### Putative involvement of the dysregulated circRNA hosting genes in viral carcinogenesis and cellular antiviral response

**Figure 6:**
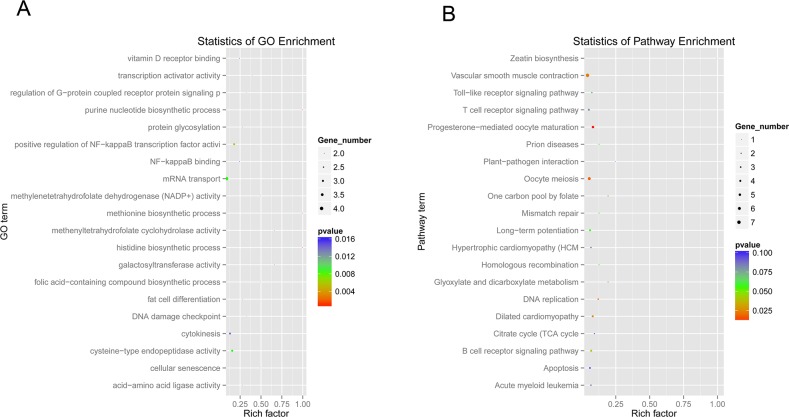
GO function and KEGG pathway enrichment analysis of dysregulated circRNA hosting genes **(A)** Gene ontology and KEGG pathway analysis of dysregulated circRNA hosting genes. The *x*-axis shows the rich factor, and the *y*-axis shows the GO term. The point size represents the number of genes enriched in a particular GO term. **(B)** The 20 most enriched KEGG pathways based on dysregulated circRNA hosting genes during SV40 infection. The *x*-axis shows the rich factor, and the *y*-axis shows the pathway names. The point size represents the number of genes enriched in a particular pathway.

As pivotal gene regulators in eukaryotes, the function of circRNAs still remained largely unknown. The analysis of the dysregulated circRNA hosting genes provided valuable clues for the circRNA functions. Based on the assumption that the function of circRNAs may be related to their parent genes, GO function enrichment and KEGG pathway analysis for dysregulated circRNA hosting genes were performed in this study to gain insight into the functions of the circRNAs. The GO analysis revealed that diverse biological processes, including cellular senescence (GO:0090398), positive regulation of NF-kappaB (nuclear factor kappa B) transcription factor activity (GO:0051092), mRNA transport (GO:0051028), DNA damage checkpoint (GO:0000077), regulation of G-protein coupled receptor protein signaling pathway (GO:0008277), cytokinesis (GO:0000910), and so on, were enriched (Figure 6A and Supplementary Table 4). This suggests that the enriched genes might be involved in cellular response to SV40 infection by alternation of dysregulated circRNA hosting genes. Furthermore, the KEGG pathway analysis revealed that a large number of enriched genes were mainly involved in carcinogenic signaling pathways, such as DNA mismatch repair (ko03430), acute myeloid leukemia (ko05221), p53 signaling pathway (ko04115), Wnt signaling pathway (ko04310), TGF-beta (transforming growth factor-β) signaling pathway (ko04350), pathways in cancer (ko05200) and so on (Figure 6B). These enriched carcinogenic signaling pathways might be affected by the SV40 large T antigen. Mounting evidence showed that SV40 large T antigen, a viral oncoprotein, could transform and immortalize many cell lines [1, 2, 3], thus potentially impacting these oncogenic signaling pathways. Additionally, other dysregulated circRNA hosting genes were enriched in cellular antiviral signaling pathways, such as Toll-like receptor signaling pathway (ko04620), Jak-STAT signaling pathway (ko04630), RIG-I-like receptor signaling pathway (ko04622), NOD-like receptor signaling pathway (ko04621), B cell receptor signaling pathway (ko04662), and T cell receptor signaling pathway (ko04660) (Figure 6B and Supplementary Table 5), thus suggesting that these host antiviral signaling pathways could be manipulated by SV40 to achieve immune escape. These results are in agreement with the notion that viruses can hijack diverse host signaling pathways, such as NF-κB, (mitogen-activated protein kinase) MAPK and JAK-STAT signaling pathway to support viral replication [33, 34, 35].

### Putative function of circRNAs acting as miRNA sponges or decoys

**Figure 7:**
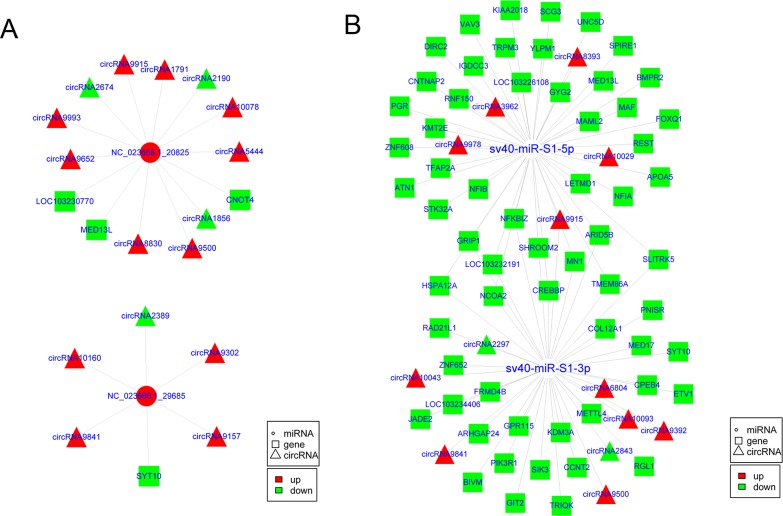
CircRNAs potentially regulated cellular genes by competitively binding cellular and viral miRNAs **(A)** The circRNA–miRNA–mRNA regulatory circuit mediated by cellular miRNAs upon SV40 infection in Vero cells. **(B)** The circRNA–miRNA–mRNA regulatory circuit mediated by viral miRNAs upon SV40 infection in Vero cells.

As miRNA “decoys” or miRNA “sponges”, circRNAs represent a new layer of gene regulation. They act as ceRNAs and indirectly regulate genes by competitively binding miRNAs with genes [36, 37]. Therefore, an integrated analysis of circRNAs, miRNAs, and genes was performed to gain insight into the function of the circRNAs. First, multiple differentially expressed miRNAs and genes associated with SV40 infection were obtained (Supplementary Tables 6 and 7) based on the whole-transcriptome sequencing and small RNA sequencing. Next, the co-expression analysis based on the Pearson correlation coefficient (*r*) between circRNAs and mRNAs, and binding site analysis of miRNAs in circRNAs and mRNAs were performed. This study identified 20 highly confident circRNA–mRNA pairs (*r* > 0.85 or *r* < -0.85, *cP* value < 0.05) (Supplementary Table 8). They functioned as SV40 infection-related circRNA–miRNA–gene regulatory circuits and might be involved in cellular responses to SV40 infection. As shown in Figure 7A, five circRNAs, including circRNA2389, circRNA10160, circRNA9302, circRNA9841, and circRNA9157, indirectly regulated mRNA transcript XM_007968135.1 (*SYT10*) by competitively binding to cellular miRNA NC_023666.1_29685. Similarly, seven circRNAs, including circRNA1856, circRNA2190, circRNA5444, circRNA9500, circRNA9652, circRNA9915, and circRNA9993, indirectly regulated mRNA transcript XM_007983021.1 (*CNOT4*) by competitively binding to cellular miRNA NC_023658.1_20825. Moreover, seven circRNAs, including circRNA10078, circRNA1791, circRNA8830, circRNA9500, circRNA9652, circRNA9915, and circRNA9993, indirectly regulated mRNA transcript XM_008004842.1 (*MED13L*) by sharing cellular miRNA NC_023658.1_20825. Notably, these results further suggested that a virus can exploit cellular miRNA–mediated gene regulatory network via ceRNA effect to aid viral infection and survive by fine-tuning of host gene expression, which is consistent with a study on primate virus herpesvirus saimiri (HVS) [38]. On the contrary, a total of 272 circRNA–mRNA interaction pairs mediated by viral sv40-miR-S1-5p/3 were further identified (*r* > 0.9 or *r* < -0.9, *cP* value < 0.05) (Supplementary Table 9). This implied that a total of 13 circRNAs indirectly regulated 62 mRNA transcripts via shared viral miRNA sv40-miR-S1-5p/3p (Figure 7B). Based on these results, it was highly likely that viruses may exploit the host gene regulatory network to promote viral infection and survival. Taken together, these results provided new clues to understand the molecular mechanisms of host–virus interaction and viral pathogenesis.

## DISCUSSION

CircRNAs, produced by RNA backsplicing, are highly prevalent in the eukaryotic transcriptome. They have been recently identified as a naturally occurring family of RNAs with regulatory potential. As they can bind to miRNAs, they are called “miRNA sponge” [28, 39]. Mounting evidence suggested that circRNAs are involved the progression of disease progression [40, 41, 42]. Especially, the crucial roles and functions of circRNAs in cancer have permanently altered the perspectives in carcinogenesis and cancer progression [43, 44, 45]. However, the expression characterization and function of circRNAs in tumor virus–infected cells is still largely unknown. Additionally, although the virus-responsive RNA expression profiles have been ananalyzed in various viruses, it is poorly understood for virus-responsive circRNA transcriptome.

In this study, the DNA tumor virus SV40 was served as a model to study viral influences on cellular circRNA transcriptome. DNA tumor viruses are important research models to address the mechanisms of tumorigenesis. In some cases, they also contribute directly to cancer. Viral tumorigenesis is generally associated with viral tumor antigens. SV40 encodes three tumor antigens, including large T antigen (T antigen), small T antigen (T antigen) and 17K T antigen. As transforming proteins, each of these tumor antigens target one or more key regulatory proteins within a cell, thus altering cell's growth/survival properties [46]. As a key oncogene protein of SV40, the large T antigen can bind to the tumor suppressor proteins Rb and p53, thus relieving cellular growth–arrest mediated by these proteins [47]. Although tumor antigens are known to be involved in the induction of viral tumorigenesis, are they associated with alteration of cellular circRNA transcriptome? If not, what is the mechanism of circRNA transcriptome changes? Further research is needed to address these central questions.

The AGMK -derived Vero cells are susceptible to a broad range of viruses and have served as a commonly used mammalian continuous cell lines in virology research [48]. Thus, Vero cells were used as the first cell model to investigate the remodeling effect of DNA tumor virus SV40 on cellular circRNA transcriptome. This study, explored potential links between circRNAs and viral pathogenicity and cellular antiviral immune response. It suggested that various carcinogenic signaling pathways (e.g., p53 and Wnt signaling pathway) and immune signaling pathways (e.g., Toll-like receptor and Jak-STAT signaling pathway) could be influenced by SV40 infection. More importantly, the integrated analysis of circRNA–miRNA–gene suggested that circRNAs potentially functioned as cellular and viral miRNA decoys and regulated the expression of cellular genes indirectly upon SV40 infection. A recent study demonstrated that circRNA-mediated avian leukosis virus induced tumor formation in chickens [49]. The present study results were in agreement with the notion that circRNAs may help to mediate tumor induction and development. However, future studies will further confirm the findings through experiments on how circRNAs are involved in viral tumorigenesis and cellular immune response. In summary, the present study described the first comprehensive expression and functional profile of circRNAs in response to SV40 infection, indicating their possible involvement in viral tumorigenesis and cellular immune response. The results would be helpful for future studies in investigating the molecular functions of circRNAs in viral pathogenesis and virus–host interactions.

## MATERIALS AND METHODS

### Cell culture and viral infection

The AGMK-derived Vero cells, an established monkey kidney epithelial cell line, were obtained from the ATCC (CCL-81) and maintained in Dulbecco's modified Eagle's medium (DMEM; HyClone) supplemented with 10% fetal bovine serum (FBS; Gibco), 2mM L-glutamine, penicillin (100 U/mL), and streptomycin (100 μg/mL) at 37°C and in 5% CO_2_ atmosphere. Vero cells were mock infected or virus infected with the SV40 strain 776 (GenBank: AF316139.1) at a multiplicity of infection of five plaque-forming units per cell in a serum-free medium. After 1 h of adsorption at 37°C, the medium was replaced with complete DMEM containing 2% FBS and incubated at 37°C and in 5% CO_2_ atmosphere.

### RNA isolation, library construction and sequencing

At 72 h postinfection, the cells were harvested and the total RNA was extracted using the TRIzol reagent (Invitrogen, CA, USA) according to the manufacturer's protocol, followed by DNase I digestion (Epicentre) for 15 min at 37°C. The integrity and quality of the total RNA was evaluated using an Agilent 2100 Bioanalyzer (Agilent Technologies) and an RNA 6000 Nano Lab Chip Kit (Agilent Technologies) with the RIN number >7.0. The RNA quantity was measured using the NanoDrop 2000 (Thermo Scientific). Two types of libraries from six samples were constructed for sequencing in this study as follows:

(1) rRNA-depleted RNA library: The rRNA was depleted in the total RNA (5 μg) using a Ribo-Zero Gold Kit (Epicentre). After purification, the rRNA-depleted RNA was fragmented using an RNA fragmentation kit (Ambion). Then, cDNA libraries were constructed using the Illumina standard mRNA-Seq Prep Kit (Illumina, CA, USA) following the manufacturer's recommendations. The prepared libraries were sequenced on an Illumina Hiseq 4000 platform (LC Sciences, Hangzhou, China), and 2 × 150 bp paired-end reads (PE150) were generated according to the Illumina's standard protocol. All Illumina sequencing raw and processed data were submitted to the Gene Expression Omnibus (GEO) database (http://www.ncbi.nlm.nih.gov/geo/) under the accession number GSE93555. (2) Small RNA library: Approximately 2.5 μg of total RNA was used to prepare a small RNA library using the stranded Illumina Truseq Small RNA Preparation Kit (Illumina, San Diego, USA) following the manufacturer's recommendations. The purified cDNA libraries were sequenced on an Illumina Hiseq 2500 platform (LC Sciences). Single-end sequencing (50 bp) reads were obtained using the Illumina's Sequencing Control Studio software version 2.8 (SCS v2.8). Then, the sequencing reads were conducted for real-time sequencing image analysis and base-calling using Illumina's Real-Time Analysis version 1.8.70 (LC Sciences). All Illumina sequencing raw and processed data were submitted to the GEO database (http://www.ncbi.nlm.nih.gov/geo/) under the accession number GSE93350.

### Reads mapping, annotation, and transcriptome assembly

For mapping of RNA-seq reads, the reference genome and genome annotation GTF files of the AGM (*Chlorocebus aethiops*) were downloaded from the National Center for Biotechnology Information (NCBI) GenBank under assembly accession number GCA_000409795.2. Raw reads were obtained and assessed for quality using FastQC (http://www.bioinformatics.babraham.ac.uk/projects/fastqc/). The reads containing adapter, poly-N, and low-quality reads were removed using Cutadapt [50] to obtain clean reads of high quality. For a circRNA analysis, clean paired-end RNA-seq reads (150 bp long) were aligned to the AGM reference genome using TopHat (v2.1.0) [51] (Tophat2, -N 2 -r 50 --library-type fr-firststrand --mate-std-dev20 -a 4 -i 70 -I 40000 --read-edit-dist 2 --min-segment-intron 70 --max-segment-intron 40000 -p 26) for linear transcripts. The mapped reads were assembled into known and novel linear transcripts using Cufflinks (v2.1.1) [52] (Cufflinks2, -I 40000 --max-bundle-frags 1000000 -p 26 -u -L CUFF). All transcripts were pooled and merged to generate final transcriptome using Cuffmerge (v 2.1.1) [52] (Cuffmerge2, -p 26). In contrast, the unmapped reads were aligned to the AGM reference genome for fusion transcripts using TopHat-Fusion (v2.1.0) [51] (Tophat-Fusion2, -N 2 --library-type fr-firststrand -a 4 -i 70 -I 40000 --read-edit-dist 2 --min-segment-intron 70 --max-segment-intron 40000 -p 26 --fusion-search --no-coverage-search --bowtie1). Fusion transcripts from TopHat-Fusion and transcripts from the assembled linear RNAs were analyzed using the CIRCexplorer algorithm [26] to identify candidate circRNAs. The transcripts that met the following criteria were identified as candidate circRNAs: (1) both ends of the splice site, GU/AG; (2) mismatch, ≤ 2; (3) backspliced junction reads, ≥1; and (4) distance between two splice sites, ≤100 KB. The low-confidence back-spliced junction reads were filtered by the computational pipeline. The number of reads spanning the backsplicing junction was used to quantify the expression of circRNA. Hosting genes producing individual circRNAs were identified by matching the genomic location of circRNAs with the location of genes detected by TopHat/Cufflinks using BEDtools [53]. For the mRNA analysis, the AGM reference transcripts were downloaded from the NCBI under the assembly accession number GCA_000409795.2. Transcripts were considered novel if they did not overlap with those annotated. For the miRNA analysis, all raw reads were first subjected to the Illumina pipeline filter and then processed using the ACGT101-miR program [54] (LC Sciences, TX, USA) to remove adapter dimers, low complexity and junk sequences. The rhesus monkey was selected as miRNA annotation reference, because of the lack of miRNA data for the AGM and because rhesus monkey was the closest species in evolution with AGM and had available miRNA data. Subsequently, clean reads were aligned to the AGM reference genome, and the unaligned reads were subjected to the BLAST search against Rfam v.10.1 and GenBank database (E-value < 0.01) to filter out rRNA, tRNA, small nuclear RNAs (snRNAs), and small nucleolar RNAs (snoRNAs). The remaining reads were aligned to the miRBase v.21 database [55] using RepeatMasker and bowtie [56] to identify known miRNAs. The unannotated reads were used to predict novel miRNAs using miRDeep2 [57].

### Differential expression analysis of transcripts

The aligned read files were processed with Cufflinks [52], which uses the normalized RNA-seq fragment counts to measure the relative abundances of transcripts. Cuffdiff (v2.1.1) [52] was used to calculate the FPKM values of circRNAs and mRNAs in all the samples. The differential expression analysis of circRNAs was conducted using a two-tailed Student *t* test and the SPSS Statistics v19.0 software package (IBM, NY, USA). The false discovery rate (FDR) was calculated to correct the *P* value, and its threshold was set at less than 0.05. The circRNAs were considered significantly differentially expressed only when the log_2_ fold change was ≥ 1 or ≤ −1 and *P* < 0.05.

The differential mRNA expression analysis was conducted using the CuffDiff program (Cuffdiff2, -p 26 --FDR 0.05) of Cufflinks. Moreover, their read counts were normalized to tags per million counts (TPM) to quantify the expression level of miRNAs. The TPM was calculated as follows: normalized expression, TPM = (actual miRNA count/number of total clean read) × 10^6^. The differential miRNA expression analysis between SV40-infected and uninfected cells was based on their raw reads using the DESeq package [58]. The binding sites of miRNAs in their targets were analyzed using miRanda (v3.3a) [59] with the parameters score of ≥150 and △G ≤ -30 kcal/mol.

### Validation of circRNAs by RT-PCR and Sanger sequencing

The total RNA from Vero cells was isolated using the TRIzol reagent (Invitrogen, CA, USA). Then, 2 μg of total RNA was reverse transcribed using the GoScript Reverse Transcription System (Promega, WI, USA) following the manufacturer's protocol. The reaction mixtures were incubated for 5 min at 25°C, followed by 1 h at 42°C and 15 min at 70°C to inactivate the reverse transcription (RT) enzyme. Moreover, genomic DNA was isolated from Vero cells using the Wizard SV Genomic DNA Purification System (Promega, WI, USA) following the manufacturer's protocol. Divergent primers that could amplify the circRNA, but not linear RNA, and convergent primers that could amplify the linear RNA, but not circRNA, were designed using the Primer Premier 5 software [60]. Monkey glyceraldehyde-3-phosphate dehydrogenase (*GAPDH*) was used as internal an control. For the PCR analysis, 1 μL of cDNA or 0.5 μg of genomic DNA was used with 500nM of divergent or convergent primers (Supplementary Table 10) (Invitrogen Co., Ltd., Shanghai, China) in a reaction mixture of 25 μL (TaKaRa, Dalian, China) containing 2.5 μL of 10 × Ex Taq Buffer (Mg^2+^ Plus), 4 μL of dNTP mixture (2.5mM), 2μM of divergent or convergent primers, and 0.625 units of TaKaRa Ex Taq DNA polymerase. PCR amplification was carried out on a Bio-Rad C1000 touch thermal cyclers (Bio-Rad) using the following program: 3 min at 95°C, followed by 40 cycles of denaturing for 15 s at 95°C, annealing for 15 s at 40°C, and extension for 15 s at 72°C and then 72°C for 10 min. Genomic DNA was served as a negative control. Then, the PCR products were visualized after gel electrophoresis in 2% ethidium bromide–stained agarose gel. The PCR products were subjected to standard Sanger sequencing using custom-designed primers to confirm the PCR results.

### RNase R resistance analysis of circRNAs

The total RNA (4 μg) from the Vero cells was treated with RNase R (RNR07250, Epicentre) or nuclease-free water (mock control) and incubated for 30 min at 37°C. The digested RNA was isolated using phenol–chloroform–isoamyl alcohol (25:24:1) (Life Science) and then precipitated using precooled isopropanol (Sinopharm Chemical Reagent Co., Ltd, China). Then, the treated RNAs were reverse transcribed with random primers using SuperScript III (Invitrogen) following the manufacturer's protocol. The reaction mixtures were incubated for 10 min at 25°C, followed by 30 min at 50°C and 5 min at 85°C to inactivate the RT enzyme. The cDNA was then used for the PCR analysis as described earlier.

### GO and KEGG pathway analysis

The dysregulated circRNA hosting genes were mapped to the terms in the GO database to analyze functional significance. The dysregulated circRNA hosting genes were also mapped to terms in the KEGG pathway database for pathway analysis.

### Integrated analysis of circRNA–miRNA–gene

CircRNA–miRNA–gene interaction networks were constructed as follows: (1) RNA co-expression analysis: Pearson correlation coefficient (*r*) and correlation *P* value (*cP* value) were used to assess a co-expression relationship between circRNAs and mRNAs using an in-house MATLAB script. The threshold *r* > 0.85 or *r* < -0.85 with *cP* value < 0.05 was considered as an strong correlation. The circRNA–mRNA pairs with a strong correlation (*r* > 0.85 or *r* < -0.85, *cP* value < 0.05) were selected for further analysis. (2) miRNA binding site analysis: The strongly co-expressed circRNA–mRNA pairs, were predicted for putative miRNA binding sites using the software miRanda 3.3a (-sc 140 -en -10 -scale 4 -strict -out). (3) A correlation analysis between miRNA and mRNA: Pearson correlation coefficient (*r*) between miRNA and mRNA was calculated. The functional circRNA–miRNA–mRNA pairs possessing a strongly negative correlation between miRNA and mRNA (*r* < -0.85 and *cP* < 0.05) were further used to construct a circRNA–miRNA–mRNA interaction network. The network graphs were constructed and visualized using Cytoscape (v3.4.0) [61].

### Data processing

Data processing, including the quantile normalization and expression analysis, was performed using the R software package (v3.3.3).

## SUPPLEMENTARY MATERIALS FIGURE AND TABLES






















