# Supplementary material for: Unique expression signatures of circular RNAs in response to DNA tumor virus SV40 infection
Source: Oncotarget. 2017 Oct 9;8(58):98609–22. doi: 10.18632/oncotarget.21694 (PMC5716754; doi:10.18632/oncotarget.21694)
Supplement: Supplementary file 1 [file oncotarget-08-98609-s001.pdf]

Unique expression signatures of circular RNAs in response to DNA tumor virus SV40 infection

SUPPLEMENTARY MATERIALS

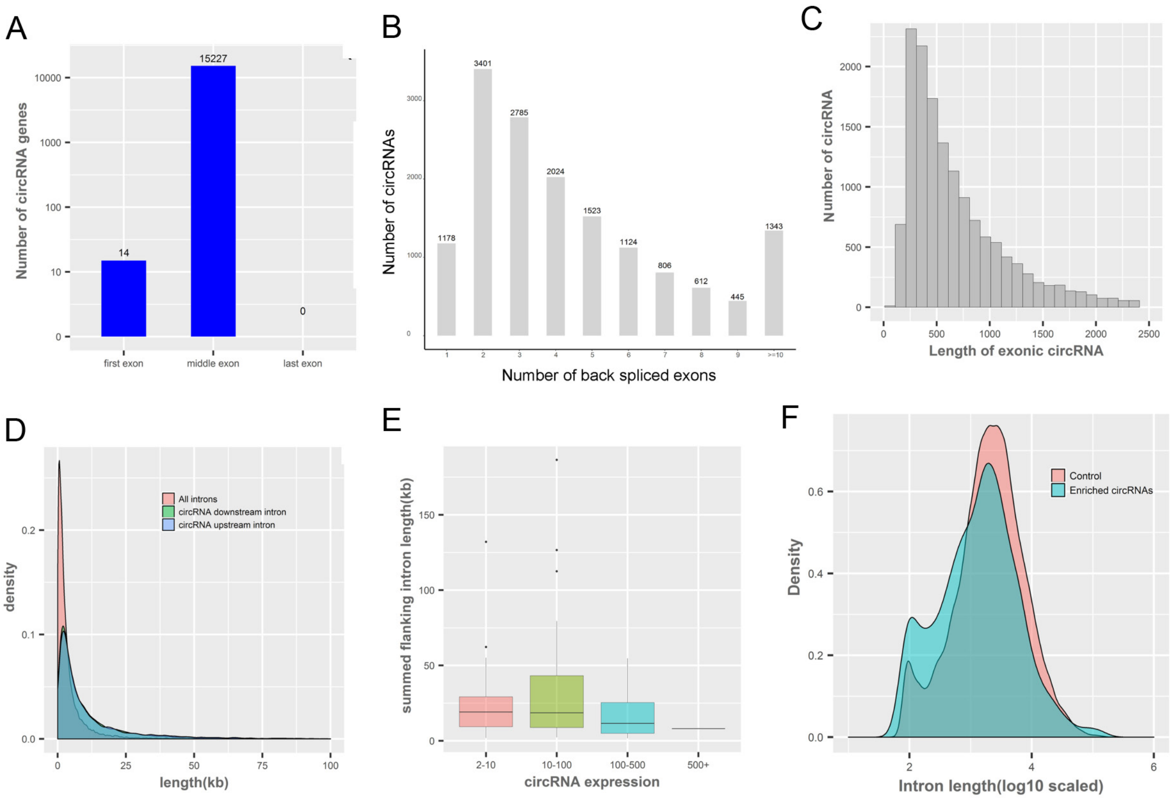

Supplementary Figure 1: Genomic features of circRNAs. (A) The location of back-spliced exons in their parental genes. Nearly all (about 99.9%) back-spliced exons were located in the middle of their hosting genes, whereas 14 were in the first exon and none were in the last exon. **(B)** The number distribution of back-spliced exons. The vast majority of circRNAs (about 90%) contained multiple back-spliced exons. **(C)** The length distribution of exonic circRNAs. **(D)** The length distributions of flanking introns of the 15,241 circRNAs. **(E)** The distribution of flanking intron lengths (sum of 5′ and 3′ flanking introns) for exons from circRNAs. **(F)** A density plot of the flanking intron length of highly abundant circRNAs (green) and low-abundance circRNAs (red).

Supplementary Table 1: Results of RNA-seq reads mapping to reference genome.

See Supplementary File 1

Supplementary Table 2: Annotation, expression, and isoforms of the circRNAs and the structural relationship with their parental genes and their flanking introns.

See Supplementary File 2

Supplementary Table 3: Differentially expressed circRNAs.

See Supplementary File 3

Supplementary Table 4: Gene ontology functional analysis of dysregulated circRNAhosting genes.

See Supplementary File 4

Supplementary Table 5: KEGG pathway analysis of dysregulated circRNA hosting genes.

See Supplementary File 5

Supplementary Table 6: Differentially expressed miRNAs induced by SV40.

See Supplementary File 6

Supplementary Table 7: Differentially expressed genes induced by SV40.

See Supplementary File 7

Supplementary Table 8: CircRNA–mRNA interaction pairs mediated by shared cellular miRNAs.

See Supplementary File 8

Supplementary Table 9: CircRNA–mRNA interaction pairs mediated by shared viral SV40-miR-S1-5p/3p.

See Supplementary File 9

Supplementary Table 10: Primers of circRNAs and their primers used for RT-PCR and Sanger sequencing.

See Supplementary File 10
