# Supplementary material for: Unique expression signatures of circular RNAs in response to DNA tumor virus SV40 infection
Source: Oncotarget. 2017 Oct 9;8(58):98609–22. doi: 10.18632/oncotarget.21694 (PMC5716754; doi:10.18632/oncotarget.21694)
Supplement: Supplementary file 11 [file oncotarget-08-98609-s011.doc]

**Supplementary Table 10: Primers of circRNAs and their primers used for RT-PCR and Sanger sequencing**

| **Primers** | **Sequence (5'-3')** | **Product (bp)** |
| --- | --- | --- |
| circRNA1273-DIV-F | CTACCTGCTGAAATTGGTGA | 289 |
| circRNA1273-DIV-R | ACTGGCTCGAAGAAGTAATC |
| circRNA1273-CON-F | AAAGACGGAAAGAAGGACTC | 454 |
| circRNA1273-CON-R | TAAAGCGAAGGTAAAGAGTG |
| circRNA1168-DIV-F | ATCACCAGCCAAAGAAATCA | 217 |
| circRNA1168-DIV-R | TCATAAGGTGCATCTGAAGT |
| circRNA1168-CON-F | AGTTTGCTCCCACAGAATAT | 179 |
| circRNA1168-CON-R | TCAGACTGTAGCGTAAATGG |
| circRNA1005-DIV-F | ATCCTGTCTCACGAAGAACA | 163 |
| circRNA1005-DIV-R | TCTGAATCTATTGGATGACC |
| circRNA1005-CON-F | ACACGGATACAACATTAGCA | 163 |
| circRNA1005-CON-R | GGAGACATTACAGATGAGGC |
| circRNA1040-DIV-F | TCAGACAAAGGACGAGATGG | 228 |
| circRNA1040-DIV-R | GAAGAAGGAGGGCTGTTGAG |
| circRNA1040-CON-F | GCCCTCCTTCTTCTATCTAC | 194 |
| circRNA1040-CON-R | CACCCTCTTTACTTCCAACT |
| ciRNA23-DIV-F | GCTCAGCACCACTCCTCATG | 179 |
| ciRNA23-DIV-R | CCAACTCCTAACCTCCCACA |
| ciRNA23-CON-F | GCGAGCTATGGGCCAAGAGT | 115 |
| ciRNA23-CON-R | GGCAGCCAGGTGGTATGTAA |
| circRNA1013-DIV-F | GGATGCTGCTTTGGATGACT | 134 |
| circRNA1013-DIV-R | TTGGCTTTCCTTCTTGGGAT |
| circRNA1013-CON-F | AGACATGGTTTCTGCTGGTG | 142 |
| circRNA1013-CON-R | TGACTTTCCCGATGGTTTAT |
| circRNA1041-DIV-F | TCAGACAAAGGACGAGATGG | 168 |
| circRNA1041-DIV-R | GAAGAAGGAGGGCTGTTGAG |
| circRNA1041-CON-F | ACAGCCCTCCTTCTTCTATC | 197 |
| circRNA1041-CON-R | AATATTAGAAACCTGTGGTTTCTCT |
| ciRNA41-DIV-F | TGAGCCCTCCACTCCTCATG | 58 |
| ciRNA41-DIV-R | CCTGTCGAGGCTGCTGTCTG |
| ciRNA41-CON-F | GGGTGGAACCCTGGTCTCCT | 102 |
| ciRNA41-CON-R | CCTTCCTGCTCAGCCTGTCG |
| GAPDH-F | GTATTGGGCGCCTGGTCACC |  |
| GAPDH-R | GGATGACCTTGCCCACAGCC |

**Sequence and back-splicing junction of circRNA candidates**

**>circRNA1273**

GAATTCTAATGAATCACTGATTGACCAGCACTATTTTACCAGTTGGAATGAATTATCAGAAATGGGCATAGTGCTTTTAGATCCAACATGTAACAGATGGATGTTACTGCATGCTGATTACTTCTTCGAGCCAGTACTTTTTTGATTGTGTAGGATCTTTGTCTCTTCATCTTTGAATTCAATTATTGGAAAATAAAAGGAGTTCATGTAGTTTTTGTCCAGGCTTGAGTCACCATGAGTAGTAGTTTAGGAAAAGAAAAAGACTCTAAAGAGAAAGATCCCAAAGTACCATCAGCCAAGGAAAGAGAAAAGGAGGCAAAAGCTTCTGGAGGTTTTGGGAAAGAGAGCAAAGAAAAAGAACCTAAGACCAAAGGGAAAGATGCCAAAGACGGAAAGAAGGACTCCAGTGCTGCCCAACCAGGGGTGGCATTTTCAGTTGACAATACGATCAAACGGCCAAACCCAGCACCTGGGACTAGAAAAAAATCCAGCAATGCAGAGGTGATTAAAGAACTCAACAAATGCCGGGAAGAGAATTCAATGCGTTTGGACTTATCCAAGAGATCTATACACATATTGCCATCATCAATCAAAGAGTTGACTCAATTAACAGAACTTTATTTATACAGTAACAAATTGCAGTCCCTCCCAGCAGAGGTGGGATGTTTAGTAAATCTCATGACATTGGCTCTAAGTGAAAATTCACTTACCAGTTTGCCTGACTCACTTGATAACTTGAAGAAGCTGCGGATGCTTGATTTACGGCATAATAAACTGAGAGAAATTCCTTCAGTGGTGTATAGGCTGGATTCTCTTACCACTCTTTACCTTCGCTTTAATCGTATAACTACTGTGGAAAAGGACATCAAAAACTTGTCAAAACTCAGCATGCTTAGCATTCGAGAGAACAAAATTAAACAACTACCTGCTGAAATTGGTGAATTATGTAACCTCATTACGCTGGATGTAGCTCACAATCAACTTGAACACCTTCCAAAGGAGATTGGAAACTGTACACAGATAACCAACCTTGACTTGCAGCACAATGAACTGCTAGATCTCCCAGATACTATAG

**back-splicing junction** GTCAAAACTCAGCATGCTTAGCATTCGAGAGAACAAAATTAAACAACTACCTGCTGAAATTGGTGAATTATGTAACCTCATTACGCTGGATGTAGCTCACAATCAACTTGAACACCTTCCAAAGGAGATTGGAAACTGTACACAGATAACCAACCTTGACTTGCAGCACAATGAACTGCTAGATCTCCCAGATACTATAGGAATTCTAATGAATCACTGATTGACCAGCACTATTTTACCAGTTGGAATGAATTATCAGAAATGGGCATAGTGCTTTTAGATCCAACATGTAACAGATGGATGTTACTGCATGCTGATTACTTCTTCGAGCCAGTACTTTTTTGATTGTGTAGGATCTTTGTCTCTTCATCTTTGAATTCAATTATTGGAAAATAAAAGG

**Divergent primer**

Forward primer 5' CTACCTGCTGAAATTGGTGA 3'

Reverse primer 5' ACTGGCTCGAAGAAGTAATC 3'

**(product 289 bp)** CTACCTGCTGAAATTGGTGAATTATGTAACCTCATTACGCTGGATGTAGCTCACAATCAACTTGAACACCTTCCAAAGGAGATTGGAAACTGTACACAGATAACCAACCTTGACTTGCAGCACAATGAACTGCTAGATCTCCCAGATACTATAGGAATTCTAATGAATCACTGATTGACCAGCACTATTTTACCAGTTGGAATGAATTATCAGAAATGGGCATAGTGCTTTTAGATCCAACATGTAACAGATGGATGTTACTGCATGCTGATTACTTCTTCGAGCCAGT

**>circRNA1168**

GGACCAGTTACTCTCCACAAGAAAATTCACACAACCACAGTGCTCTTCATAGTTCAAATTCACATTCTTCTAATCCAAGCAATAACCCAAGCAAAACTTCAGATGCACCTTATGATTCTGCAGATGACTGGTCTGAGCATATTAGCTCTTCTGGGAAAAAGTACTACTACAATTGTCGAACAGAAGTTTCACAATGGGAAAAACCAAAAGAGTGGCTTGAAAGAGAACAGAGACAAAAAGAAGCAAACAAGATGGCAGTCAACAGCTTCCCAAAAGATAGAGATTACAGAAGAGAGGTGATGCAAGCAACAGCCACTAGTGGGTTTGCCAGTGGAATGGAAGACAAGCATTCCAGTGATGCCAGTAGTTTGCTCCCACAGAATATTTTGTCTCAAACAAGCAGACACAATGACAGAGACTACAGACTGCCAAGAGCAGAGACTCACAGTAGTTCTACGCCAGTACAGCACCCCATCAAACCAGTGGTTCATCCAACTGCTACCCCAAGCACTGTTCCTTCTAGTCCATTTACGCTACAGTCTGATCACCAGCCAAAGAAATCATTTGATGCTAATGGAGCATCTACTTTATCAAAACTGCCTACACCCACATCTTCTGTCCCTGCACAGAAAACAGAAAGAAAAG

**back-splicing junction** CAGTAGTTCTACGCCAGTACAGCACCCCATCAAACCAGTGGTTCATCCAACTGCTACCCCAAGCACTGTTCCTTCTAGTCCATTTACGCTACAGTCTGATCACCAGCCAAAGAAATCATTTGATGCTAATGGAGCATCTACTTTATCAAAACTGCCTACACCCACATCTTCTGTCCCTGCACAGAAAACAGAAAGAAAAGGGACCAGTTACTCTCCACAAGAAAATTCACACAACCACAGTGCTCTTCATAGTTCAAATTCACATTCTTCTAATCCAAGCAATAACCCAAGCAAAACTTCAGATGCACCTTATGATTCTGCAGATGACTGGTCTGAGCATATTAGCTCTTCTGGGAAAAAGTACTACTACAATTGTCGAACAGAAGTTTCACAATGGGAA

**Divergent primer**

Forward primer 5' ATCACCAGCCAAAGAAATCA 3'

Reverse primer 5' TCATAAGGTGCATCTGAAGT 3'

**(product 217 bp)**

ATCACCAGCCAAAGAAATCATTTGATGCTAATGGAGCATCTACTTTATCAAAACTGCCTACACCCACATCTTCTGTCCCTGCACAGAAAACAGAAAGAAAAGGGACCAGTTACTCTCCACAAGAAAATTCACACAACCACAGTGCTCTTCATAGTTCAAATTCACATTCTTCTAATCCAAGCAATAACCCAAGCAAAACTTCAGATGCACCTTATGA

**>circRNA1005**

GTCGAATGAGTGATTTGAGTGTAATTGGTCATCCAATAGATTCAGAATCTAAAGAAGATGAACCTTGTAGTGAAGAAACAGATCCAGTGCATGATCTAATGGCTGAAATTTTACCTGAATTCCCTGACATAATTGAAATAGACCTATACCACAGTGAAGAAAATGAAGAAGAAGAAGAGTGTGCAAATGCTACTGATGTGACAACCACCCCATCTGTACAGTACATAAATGGGAAGCATCTAGTTACCACTGTGCCCAAGGACCCAGAAGCTGCAGAAGCTAGGCGTGGCCAGTTTGAAAGTGTTGCACCTTCTCAAAATTTCTCGGACAGCTCTGAAAGTGATACTCGTCCATTTGTAATAGCTGAAACAGAATTGTCTACTGCTGTGCAACCTAATGGATCTACAGAAACAACTGAATCTCTTGAACTTACATGGAAGCCTGAGACTTACCCTGAAACATCAGAACATTTTTCAGGTGGTGAGCCTGATGTTTTCCCCACAGTCCCATTCCATGAGGAATTTGAAAGTGGAACAGTCATAAAGGGGGCAGAATCAGTCACAGAGAGAGATACTGAAGTTGGTCATCAGGCACATGAACACACTGAACCCGTGTCTCTGTTTCCTGAAGAATCTTCAGGAGAGATTGCCATTGACCAAGAATCTCAGAAAATAGCCTTTGCAAGGCCTACAGAAGTAACATTTGGTGAAGAGGTAGAAAAAAGTACTTCTGTCACATACACTCCTACTGTAGTTCCAAGTTCTGCATCAGCATATGTTTCAGAGGAAAAAGCAGTTACCCTAATAGGAAATCTTTGGCCAGATGACCTGTTGTCTACCAAAGAAAGCTGGGTAGAAGCAACTCCTAGACAAGTTGTAGAGCTCTCAGGCAGTTCTTCAATTCCAGTTACAGAAGGCTCTGGAGAAGCAGAAGAAGATGAAGATACAAAGTTCACCATGGTAACTGATTTATCACAGAGAAATACTACTGATACACTCGTTACTTTAGACACTAGCAGGATAATCACAGAAAGCTTTTTTGAGGTTCCTGCAACCACCATTTATTCAGTTTCTGAACAACCTTCTGCAAAAGTGGTGCCTACCAAGTTTGTCAGTGAAACAGACACTTCTGAGTGGATTTCTAGTACCTCTGCTGAGGAAGAGAAAAGGAAGGAGGAGGAGGGAACTACAGGTACGGCTTCCACAGTTGAGGTATATTCACCTACACAGAGATCGGATCAATTAATTTTACCCTCTGAATTAGAAAGTTCAAATGTAGTTGCATCTAGTGATTCAGGTACCAGGAAAAGTTTTATGTCCTTGACAACACCAACACAGTCTGAAAGGGAAATGACAGATTCTACTCTTGTCTTTACAGAAACAAATACATTAGAAAATTTGGAGGCACAGACCACTGAGCACAGCAGTATGCGTCAACCTGGGGTTCAGGAAGGGCTGACTACTCTCCCAGGTAGTCCTGCCTCTCTCTTTATGGAGCAGGGCTCTGGAGAAGCTGCTGCTGACCCAGAAACCACCACTGTTTCTTCATTTTCATTAAATTTAGAGTATGAAATTCAAGCCAAAAAGGAAGCAGCTGGCACTTTGTCTCCATATGTGGAAACTACATTCTCCACTGAGCCAACAGGACTGGTTATGAGTACAGTAATGGACAGAGAAGTTGCTGAAAATATAAGCCAAACATCCAGGGAAATATTGATTTCAGAACGATTAGGAGAACCAAATCATGGGGCAGAAATAAGGGGCTTTTCCACAGGTTTTCCTTTGGAGGAGGATTTTAGCGGTGACTTTAAAGAATACTCAACAGTGTCTCATCCCATAGCAAAAGAAGAAACAGTAATGATGGAAGGCTCTGGAGATGCAGCATTTAGGGATACCCAGACTTCACCATCTACAGTACCTACTTCAGTTCACATCAGTCACATATCTGACTCAGAAGGACCCAGTAGCACCATGGTCAGCACTTCAGCCTTCCCCTGGGAAGAGTTTACATCCTCAGCTGAGGGCTCAGGTGAGCAACTGGTCACAGTCAGCAGCTCTGTTGATCCAGTGCTTCCCAGTGCTATGGGAAGGTTTTCTGGTACAGCTTCCTCCATTATTGACGAAGGATTGGGAGAAGTGGATACTGTCAATGAAATTGATAGAAGATCCACCATTTTACCAACAGAAGTGGAAGGTACGAAAGCCCCAGTAGAAAAGGAGGAAGTAAAGGTCAGTGGCACAATTTCAACAAACTTTCCCCAAACTATGGAGCCAGCCAAATTATGGTCTAGGCAAGAAGTCAACCCTGAAAGACAAGAAATTGAAAGTGAAACAACATCAGAGGAACAAATTCAAGAAGAAAAGTCTTTTGAATCCCCCCAAAACTCTCCTGCAACAGAACAAACAATCTTTGATTCACAGACATTTACTGAAACTGAACTCAAAACCACAGGTTATTCTGTACTAACGACAAAGAAAACTTACAGCGATGATAAAGAAATGGAGGAGGAAGGCACTTCCTTAGCTAACATGTCTACTCCAGATCCAGTTGCAAATGGCATGGAATCTTTTACAACTCTCCCTGAAGCTACTGAAAAGTCACATTTTTTCTTAGCTCCTGCCTTAGTGACTGAATCTATACCAGCTGAACATGTAGTCACAGATTCACCAATCGAAGAGGAAGAAAGTACAAAACATTTTCCCAAAGGCATGAGACCAACAATTCAAGAGTTAGATACTGAGCTCTTATTCTCTGGACTGGGATCAGGAGAAGAAGTTTTACCTACTCTACCAACAAAGTCAGTGAATGTTACTGAAGTGGAACAGATTAGGAACACATTCTATCCCCACAGTTCTCAAGTGGAAAGTACCTCAAGTGACAAAACTGAAGACTTTAACAGAATGGAAAATGTGGCAAAAGAAGTTGGACCACTCGTGTCTCAGACAGACATCTTTGAAGGTAATGAGTCAGTAACCAGCACAACCTTAATAGAAATTTTAAGTGACACTGGAGCAGAAGGACCCACGGTGGCACCTCTCCCTTTCTCCGCGAACATCGGACATCCGCAAAATCAGACTCTCAGATGGGCAGAAGAAATCCAGACTAGTAGACCACAAACCATAACTGAACAAGACTCTAACAAGAATTCTTCAACAGCAGAAATTAACGAAACAACAACCTCATCCACTGATTTTCTGGCTAGAGTTTATGGGTTTGAAATGGCCAAAGAATTTGTTACATCAGCACCAAAACCATCTGATATGTTTTATGAACCTTCTGGAGAAGGATCTGGAGAAGCGGATATTGTTGATTCATTTCACACTTCTGCAACTACTCAGGCAATCAGACAAGAAAGCAGCACCACGTTTGTTTCTGATGGGTCACTGGAAAAACATCCTGAGGTGCCAAGCACTAAAGCTGTTACTGCTGATGGATTCCCAACAGTTTCAGCGATGCTGCCTTTTCATTCAGAGCAGAACAAAAGCTCCCCTAATCCAACTAGCACACTGTCAAATACAGTGTCATATGAGAGGTCCACAGACGGTAGTTTCCAAGACCATTTCAGGGAATTTGAGGATTCCACCTTAAAACCTAACAGAAAAAAACCCAGCGAAAATATTATTATAGACCTGGACAAAGAGGACAAGGATTTGATATTGACAATTACAGAGAGTACCATCCTTGAGATTCTACCTGAGCTGACATCAGATAAAAATACTATCATAGATATTGATCATACTAAACCTGTATATGAAGATATTCTTGGAATGCAAACAGATATAGATCCAGAGGTACCATCAGAACCACATGACAGTAATGATGAAAGTAATGATGACAGCACTCAAGTTCAAGAGACCTATGAAGCAGCTGTCAACCTTTCTTTAACGGAGGAAACATTTGAAGGCTCTGGTGATGTTCTGGCTAGCTACACTCAGGCAACACATGATGAATCAATGACTTATGAAGATAGAAGCCAACTAGATCACATGGACTTTAACTTCACAACTGGGATCCCTGCTCCTAGCACAGAAACAGAACTAGACATTTTACTTCCCACGGCAACATCTCTGCCAATTCCTCGTAAGTCTGCCACAGTTATTCCAGAGACTGAAAAAATAAAAGCTGAAGCAAAAGCCCTGGATGACATGTTTGAATCAAGCACTTTGTCTGATGGTCAAGCTATTGCAGACCAAAGTGAAATAATACCAACATTGGGCCAATTTGAAAGGACCCAGGAGGAGTATGAAGACAAAAAACATGCGGGTCCTTCTTTTCAGCCAGAATTCTCTTCAGGAGTCGAGGAGGCATTAGTAGATCATACTCCCTATCTAAGTATTGCTACTACTCACCTTGTGGATGAGAGTTTAACAGAGGTGCCTAATGTGATGGAAGGATCCAATGCCCCATATTACACGGATACAACATTAGCAGTTTCAGCATTTGCAAAGTTGTCTTCTCAGACACCGTCATCTCCGCTCACTATCTACTCAGGCAGTGAAGCCTCTGGACACACAGAGATCCCCCAGCCCAGTGCTCTGCCAGGAATCGATGTCGCCTCATCTGTAATGTCTCCAGAGGATTCTTTTAAGGAAATTCATGTAAATATTGAAGCGACTTTCAAACCATCAAGTGAGGAATACCTTCACATAACTGAGCCTCCCTCTATATCTCCTGACACAAAATTAGAACCTTCAGAAGAGGATGGTAAGCCTGAGTTATTAGAAGAAACGGAAGCTTCTCCCACAGAACTTATTGCTGTGGAAGGAACTGAGATTCTCCAAGATTTCCAAAACAAAACCGATGGTCAAGTTTCTGGAGAAGCAATCAAGATGTTTCCCACCATTAAAACACCCAAAGCTGGAACTGTTATTACAACTGCCAATGAAATTGAATTAGAAGGTGCTACACAGTGGCCACACTCTACTTCTGCTTCTGCCACTTACGGGGTCGAGGCAGGTGTGATGCCTTGGCTGAGTCCACAGACTTCTGAGAGGCCCACGCTTTCTTCTTCTCCAGAAATAAATCCTGAAACTCAAGCAGCTTTAATCAGAGGGCAGGATTCCACAGTAGCAGCATCAGAACAGCAAGTGGCAACGAGAATTCTTGATTCCAATAATCAGGCAACTGTAAGCCCGGTGGAATTTAATACTGAGGTTGCAACACCACCATTTTCCCTTCTGGAAACTTCTAATGAAACAGATTTCCTGATTGGCATTAATGAAGAGTCAGTGGAAGGCACGGCAATCTATTTACCAGGACCTGATCGTTGCAAAATGAACCCGTGCCTTAACGGAGGCACCTGTTATCCTACTGAAACTTCCTATGTATGCACCTGTGTGCCAGGATACAGTGGAGACCAGTGTGAACTTGATTTTGATGAATGTCACTCTAATCCCTGTCGGAATGGAGCCACTTGTGTTGATGGTTTTAACACATTCAGGTGCCTCTGCCTTCCAAGTTATGTTGGTGCACTTTGTGAACAAGACACCGAGACATGTGACTATGGCTGGCACAAATTCCAAGGGCAGTGCTACAAATACTTTGCCCATCGACGCACATGGGATGCAGCTGAACGGGAATGCCGTCTGCAGGGTGCCCATCTCACAAGCATCCTGTCTCACGAAGAACAAATGTTTGTTAATCGTGTGGGCCATGACTATCAGTGGATAGGCCTTAATGACAAGATGTTTGAGCATGACTTCCGCTGGACTGATGGCAGCACACTG

**back-splicing junction** AGTGCTACAAATACTTTGCCCATCGACGCACATGGGATGCAGCTGAACGGGAATGCCGTCTGCAGGGTGCCCATCTCACAAGCATCCTGTCTCACGAAGAACAAATGTTTGTTAATCGTGTGGGCCATGACTATCAGTGGATAGGCCTTAATGACAAGATGTTTGAGCATGACTTCCGCTGGACTGATGGCAGCACACTGGTCGAATGAGTGATTTGAGTGTAATTGGTCATCCAATAGATTCAGAATCTAAAGAAGATGAACCTTGTAGTGAAGAAACAGATCCAGTGCATGATCTAATGGCTGAAATTTTACCTGAATTCCCTGACATAATTGAAATAGACCTATACCACAGTGAAGAAAATGAAGAAGAAGAAGAGTGTGCAAATGCTACTGATGTG

**Divergent primer (163)**

5' ATCCTGTCTCACGAAGAACA 3'

5' TCTGAATCTATTGGATGACC 3'

ATCCTGTCTCACGAAGAACAAATGTTTGTTAATCGTGTGGGCCATGACTATCAGTGGATAGGCCTTAATGACAAGATGTTTGAGCATGACTTCCGCTGGACTGATGGCAGCACACTGGTCGAATGAGTGATTTGAGTGTAATTGGTCATCCAATAGATTCAGA

**>circRNA1040**

AAATTATACCACTTTATGGAATGTCAAGCTACATCACCCGAGAAGACCAGTACAGCAAGCCTCCGCACAAAAAACTGAAAGACCGCCAGATCGATCGCCAGAACCGCCTCAACAGCCCTCCTTCTTCTATCTACAAAAGCAGCTGCACAACAGTATACAATGGCTACGGGAAGGGCCACAGCAGTGGAAGTGGCGGAGGCGGCAGCGGTGGTGGTCCTGGAATTAAGAAAACAGAGCGACGAGCAAGAAGCAGCCCAAAGTCGAATGATTCAGACTTGCAAGAATATGAGTTGGAAGTAAAGAGGGTGCAAGACATTCTTTCGGGAATAGAGAAACCACAGGTTTCTAATATTCAGGCGAGAGCAGTTGTGTTGTCCTGGGCTCCCCCTGTTGGACTTTCCTGTGGACCCCACAGTGGTCTTTCCTTCCCCTACAGTTATGAGGTTGCCTTATCAGACAAAGGACGAGATGGAAAATACAAGATAATTTACAGTGGAGAAGAATTAGAATGTAACCTGAAAGATCTTAGACCAGCAACAGATTATCATGTGAG

**back-splicing junction**

CAGGCGAGAGCAGTTGTGTTGTCCTGGGCTCCCCCTGTTGGACTTTCCTGTGGACCCCACAGTGGTCTTTCCTTCCCCTACAGTTATGAGGTTGCCTTATCAGACAAAGGACGAGATGGAAAATACAAGATAATTTACAGTGGAGAAGAATTAGAATGTAACCTGAAAGATCTTAGACCAGCAACAGATTATCATGTGAGAAATTATACCACTTTATGGAATGTCAAGCTACATCACCCGAGAAGACCAGTACAGCAAGCCTCCGCACAAAAAACTGAAAGACCGCCAGATCGATCGCCAGAACCGCCTCAACAGCCCTCCTTCTTCTATCTACAAAAGCAGCTGCACAACAGTATACAATGGCTACGGGAAGGGCCACAGCAGTGGAAGTGGCGGAGGC

**Divergent primer (228)**

5' TCAGACAAAGGACGAGATGG 3'

5' GAAGAAGGAGGGCTGTTGAG 3'

TCAGACAAAGGACGAGATGGAAAATACAAGATAATTTACAGTGGAGAAGAATTAGAATGTAACCTGAAAGATCTTAGACCAGCAACAGATTATCATGTGAGAAATTATACCACTTTATGGAATGTCAAGCTACATCACCCGAGAAGACCAGTACAGCAAGCCTCCGCACAAAAAACTGAAAGACCGCCAGATCGATCGCCAGAACCGCCTCAACAGCCCTCCTTCTTC

**>ciRNA23**

GCAAGTGAACCAAGAGTTCTGTATTCAGTCCTACCTTTCCCTTCAGAGCATCCTGCCCCCTCTGATTCCAGCGACACACACAGGATGGGACGTTCTCCACTGCAGGGCTTGTCTGTAGGTGTGGGAGGTTAGGAGTTGGTTTTGTCCTCTCACGTCTACCCTGAGCAGTAGTGAGTAAGCTCTGCTAATGCAGGAGTTCTAAAAATGTTTTTCTTTAAGGAAACTCCAGAGCCAGGAATATTAATTGTAGGAGTTTCTAAAACTTAACATGCAGACCAGGAATTAAGGCAGGTGTGACACAGAGAGGGGGCAGCACTGGGTGTGTCCCTACTACTCACACTGGGCTCAGACCTCTTCAGTGTCTCCTCAGGGAGTGCTGGTCTGAGTGGAGGTCATAGCCAAGATCCCTGGAGGAGCCAGGCAGAAGCCACCGTTGAAGTGGATGGTGTGGAAGGCAGCGGTGGGGGATGGGGACAGCTGACAGACTCCTCATTGTCACACTGCAGCCGTGTGTAAACCAGGGTCTGTGTGCACAACCTGCTGTGCGCTGCCAGTGTGTCTTCTCTGCCTTAGGGCAGGGGATGGCGAGCTATGGGCCAAGAGTAGTTCTTGTATTTTTCAATCAAAAGATGTGACAGTTAGATGAAATGCGTATTTCAGTGTCCAGCGATAGTTTTGTTTACATACCACCTGGCTGCCTTGTGTCACAGTGGCAGAGTTGAGCAGTGTGAAAAAGACTAGTTGGCCCTTTATAGGGCAGGCAGGTCCACTGTGGCCTGTGAGGGCCAGAGCTCTGGGCACTCGGACACTGGCAGGCCCCGGTCCCGCTGGCCAAGGCAGGAGGGTGTGTGTTTCGGGTCACTCACAGGGCTCAGCACCACTCCTCATGGCTTCCTTACTGTTTTGGCA

**back-splicing junction**

TGGCAGAGTTGAGCAGTGTGAAAAAGACTAGTTGGCCCTTTATAGGGCAGGCAGGTCCACTGTGGCCTGTGAGGGCCAGAGCTCTGGGCACTCGGACACTGGCAGGCCCCGGTCCCGCTGGCCAAGGCAGGAGGGTGTGTGTTTCGGGTCACTCACAGGGCTCAGCACCACTCCTCATGGCTTCCTTACTGTTTTGGCAGCAAGTGAACCAAGAGTTCTGTATTCAGTCCTACCTTTCCCTTCAGAGCATCCTGCCCCCTCTGATTCCAGCGACACACACAGGATGGGACGTTCTCCACTGCAGGGCTTGTCTGTAGGTGTGGGAGGTTAGGAGTTGGTTTTGTCCTCTCACGTCTACCCTGAGCAGTAGTGAGTAAGCTCTGCTAATGCAGGAGTTCTA

**Divergent primer (179)**

5' GCTCAGCACCACTCCTCATG 3'

5' CCAACTCCTAACCTCCCACA 3'

GCTCAGCACCACTCCTCATGGCTTCCTTACTGTTTTGGCAGCAAGTGAACCAAGAGTTCTGTATTCAGTCCTACCTTTCCCTTCAGAGCATCCTGCCCCCTCTGATTCCAGCGACACACACAGGATGGGACGTTCTCCACTGCAGGGCTTGTCTGTAGGTGTGGGAGGTTAGGAGTTGG

**>circRNA1013**

CTGTCTGTGGTTCATGAGAAAAAATCCCAAGAAGGAAAGCCAAAAGAACATACAGAGCAAAAAAGCCTACCCAAGCCGGCATCAGATACAGGAAGTAAGGATGCTCACAATAAAAAAGCAGTTTCCAGATCAGCTGAACAGCAGCCATCAGAGAAATCAACAGAACCAAAGACTGAACCACAAGACATGGTTTCTGCTGGTGGAGAGAGCGTTGCTGGTGTCGCTGCAACATCTGGCAAGCCAGGTGACAAGAAAAAAGAAAAGAAATCGTTAACCCCAGCTGTGCCAGTTGAATCTAAACCGGATAAACCATCGGGAAAGTCAGGCATGGATGCTGCTTTGGATGACTTAATAGATACTTTAGGAGGACCTGAAGAAATTGAAGAAGAAAATACAACGTATACTGGACCAGAAGTTTCA

**back-splicing junction**

TCGCTGCAACATCTGGCAAGCCAGGTGACAAGAAAAAAGAAAAGAAATCGTTAACCCCAGCTGTGCCAGTTGAATCTAAACCGGATAAACCATCGGGAAAGTCAGGCATGGATGCTGCTTTGGATGACTTAATAGATACTTTAGGAGGACCTGAAGAAATTGAAGAAGAAAATACAACGTATACTGGACCAGAAGTTTCACTGTCTGTGGTTCATGAGAAAAAATCCCAAGAAGGAAAGCCAAAAGAACATACAGAGCAAAAAAGCCTACCCAAGCCGGCATCAGATACAGGAAGTAAGGATGCTCACAATAAAAAAGCAGTTTCCAGATCAGCTGAACAGCAGCCATCAGAGAAATCAACAGAACCAAAGACTGAACCACAAGACATGGTTTCTGCTGG

**Divergent primer (134)**

5' GGATGCTGCTTTGGATGACT 3'

5' TTGGCTTTCCTTCTTGGGAT 3'

GGATGCTGCTTTGGATGACTTAATAGATACTTTAGGAGGACCTGAAGAAATTGAAGAAGAAAATACAACGTATACTGGACCAGAAGTTTCACTGTCTGTGGTTCATGAGAAAAAATCCCAAGAAGGAAAGCCAA

**>circRNA1041**

AAATTATACCACTTTATGGAATGTCAAGCTACATCACCCGAGAAGACCAGTACAGCAAGCCTCCGCACAAAAAACTGAAAGACCGCCAGATCGATCGCCAGAACCGCCTCAACAGCCCTCCTTCTTCTATCTACAAAAGCAGCTGCACAACAGTATACAATGGCTACGGGAAGGGCCACAGCAGTGGAAGTGGCGGAGGCGGCAGCGGTGGTGGTCCTGGAATTAAGAAAACAGAGCGACGAGCAAGAAGCAGCCCAAAGTCGAATGATTCAGACTTGCAAGAATATGAGTTGGAAGTAAAGAGGGTGCAAGACATTCTTTCGGGAATAGAGAAACCACAGGTTTCTAATATTCAGGCGAGAGCAGTTGTGTTGTCCTGGGCTCCCCCTGTTGGACTTTCCTGTGGACCCCACAGTGGTCTTTCCTTCCCCTACAGTTATGAGGTTGCCTTATCAGACAAAGGACGAGATGGAAAATACAAGATAATTTACAG

**back-splicing junction**

GAAGTAAAGAGGGTGCAAGACATTCTTTCGGGAATAGAGAAACCACAGGTTTCTAATATTCAGGCGAGAGCAGTTGTGTTGTCCTGGGCTCCCCCTGTTGGACTTTCCTGTGGACCCCACAGTGGTCTTTCCTTCCCCTACAGTTATGAGGTTGCCTTATCAGACAAAGGACGAGATGGAAAATACAAGATAATTTACAGAAATTATACCACTTTATGGAATGTCAAGCTACATCACCCGAGAAGACCAGTACAGCAAGCCTCCGCACAAAAAACTGAAAGACCGCCAGATCGATCGCCAGAACCGCCTCAACAGCCCTCCTTCTTCTATCTACAAAAGCAGCTGCACAACAGTATACAATGGCTACGGGAAGGGCCACAGCAGTGGAAGTGGCGGAGGC

**Divergent primer (168)**

5' TCAGACAAAGGACGAGATGG 3'

5' GAAGAAGGAGGGCTGTTGAG 3'

TCAGACAAAGGACGAGATGGAAAATACAAGATAATTTACAGAAATTATACCACTTTATGGAATGTCAAGCTACATCACCCGAGAAGACCAGTACAGCAAGCCTCCGCACAAAAAACTGAAAGACCGCCAGATCGATCGCCAGAACCGCCTCAACAGCCCTCCTTCTTC

**>ciRNA41**

GTACAGAGTGGAGCCATGGCAGCAGGGGTGGAACCCTGGTCTCCTGGGGTGAGCAGAGCAGGCTCCCTGTCTCATGGACTGGGCCCTGGGTAGACAGACAGCAGCCTCGACAGGCTGAGCAGGAAGGCCTGAGCCCTCCACTC

**back-splicing junction**

CTCATGGACTGGGCCCTGGGTAGACAGACAGCAGCCTCGACAGGCTGAGCAGGAAGGCCTGAGCCCTCCACTCCTCATGGACTGGGCCCTGGGTAGACAGACAGCAGCCTCGACAGGCTGAGCAGGAAGGCCTGAGCCCTCCA

**Divergent primer (58)**

5' TGAGCCCTCCACTCCTCATG 3'

5' CCTGTCGAGGCTGCTGTCTG 3'

TGAGCCCTCCACTCCTCATGGACTGGGCCCTGGGTAGACAGACAGCAGCCTCGACAGG
